# Adiponectin and Omentin Levels as Predictive Biomarkers of Preterm Birth in Patients with Gestational Diabetes Mellitus

**DOI:** 10.1155/2018/7154216

**Published:** 2018-09-18

**Authors:** Radzisław Mierzyński, Dominik Dłuski, Łukasz Nowakowski, Elżbieta Poniedziałek-Czajkowska, Bożena Leszczyńska-Gorzelak

**Affiliations:** ^1^Chair and Department of Obstetrics and Perinatology, Medical University of Lublin, Poland; ^2^II Chair and Department of Gynecology, Medical University of Lublin, Poland

## Abstract

**Objective:**

The aim of this study was to determine any changes in adiponectin and omentin levels in GDM patients who delivered at term and preterm and to evaluate whether adipokines can be useful as a clinical biomarker to predict subsequent preterm delivery.

**Patients and Methods:**

The levels of adiponectin and omentin were measured in four groups: (1) women with GDM who delivered at term (n=63); (2) women with GDM who had the symptoms of threatened preterm labor and delivered at term (n=23); (3) women with GDM and spontaneous preterm birth (before 37 completed weeks of gestation) (n=19); (4) women with physiological pregnancy (n=55).

**Results:**

In comparison with control group the median adiponectin concentrations were significantly lower in all GDM groups (10737 versus 8879; 7057; 6253 ng/ml, respectively; p<0.01). The median omentin concentrations were also significantly lower in all GDM groups in comparison with control group (469 versus 432; 357; 308 ng/ml, respectively; p<0.01). No significant differences in adiponectin and omentin levels between the GDM, preterm labor, and preterm birth groups were observed. However, there was a trend towards lower adiponectin and omentin levels in preterm birth group. The strong correlations between adiponectin and omentin levels were observed in all groups (R=0.801, p<0.001; R=0.824, p<0.001; R=0.705, p<0.001; R=0.764, respectively; p<0.001). In the univariable logistic regression model, significant correlation between omentin concentrations and preterm birth occurrence was found.

**Conclusions:**

Our findings suggest that omentin-1, rather than adiponectin, could be useful as a predictor of preterm birth in patients with gestational diabetes mellitus.

## 1. Introduction

Preterm birth (PTB) is defined as any birth before 37 completed weeks of gestation or fewer than 259 days since the first day of the last menstrual period [1]. The worldwide frequency of PTB is unchanged over two decades. Every year, an estimated 15 million infants are born preterm globally [2]. Decreasing gestational age at delivery is connected with higher infant mortality and disability risk. PTB is an important precursor to future morbidity in both developed and developing countries [3].

PTB is a syndrome with a variety of causes and can be divided into two main groups: iatrogenic preterm birth (30-35%) and spontaneous preterm birth (65-70%). The exact cause of spontaneous preterm labor and delivery can not be determined in almost one-half of all cases [4, 5]. The pathophysiology of PTB also differs between spontaneous and medically indicated births. Main causes of PTB include stress, incompetent cervix, placental ischemia, decidual hemorrhage, placental abruption, systemic or cervical maternal genital tract infections, uteroplacental insufficiency, multiple gestation, and chronic conditions such as high blood pressure, gestational diabetes, blood clotting disorders, and maternal periodontal disease [6].

Other factors are obesity or underweight before and during pregnancy, having a previous premature birth, in vitro fertilization, extreme maternal age (<17 or >35 years old), smoking cigarettes, nonwhite race, and physical injury or trauma [5].

It has been published that the risk for spontaneous preterm delivery decreases as maternal body mass index (BMI) increases. Obese women also have fewer contractions and longer cervical lengths and are more likely to deliver after their due date [7, 8]. The reason for this protective effect is not known and is understudied.

Gestational diabetes mellitus (GDM) is defined as any degree of glucose intolerance with onset or first detected during pregnancy. It is the most common metabolic disorder of pregnant women and represents one of the main problems in perinatal medicine. Prevalence of GDM may range from 5 to 20% of all pregnancies, depending on the population tested and the screening methods employed. GDM is associated with adverse maternal and neonatal outcomes. Women with GDM have also significantly elevated risk for type 2 diabetes mellitus (T2DM) in later life [9]. It is agreed that diabetes is associated with preterm birth—either spontaneous or medically indicated. However, some investigators did not notice a higher risk for PTB in patient with GDM [10], but the others reported that GDM by itself is a risk factor for PTB [11]. The results from the HAPO study that shows linear relationship between maternal hyperglycemia and increased risk of birth weight above the 90th percentile, primary cesarean delivery, premature delivery, and shoulder dystocia have been published [12].

Adipose tissue is a highly active endocrine organ and produces a number of adipokines. It has been described that the placenta produces similar adipokines as adipose tissue and that their serum levels can change during the pregnancy. It has been also reported that adipokines might play an important modulatory role in maternal-fetal adaptations and several adipocytokines have been analyzed throughout gestation and their levels have been suggested as biomarkers of pregnancy-related complications including preterm delivery [13, 14].

Adiponectin is a 244-amino-acid-long polypeptide hormone which is a member of adipokines group that modulates a number of metabolic processes. Adiponectin is almost uniquely synthesized by adipocytes but also within the intrauterine environment and stimulates the glucose uptake in skeletal muscles and decreases the hepatic glucose synthesis [15]. However, the signaling pathway of adiponectin (5′AMP-activated protein kinase) has been described as a mechanism important to the myometrium; no data are available about the adiponectin influence on regulation of uterine contractility [15].

Omentin is a novel adipokine of 313 amino acids and is mainly synthesized by visceral adipose tissue. Omentin is also expressed and secreted by the human placenta. Two high isoforms have been described: omentin-1 and omentin-2 with 83% amino acid identity. Omentin-1 is the major circulating form [16]. Omentin levels are inversely correlated with obesity and positively with adiponectin levels [17]. It has been observed that circulating maternal omentin-1 concentrations were higher in normal spontaneous term births than in preterm births. These results suggest that omentin could play an important role in the pathophysiology of preterm delivery [18].

It is possible that an alteration in the maternal endocrine or metabolic status, observed in GDM, and modification in adipokines secretion may have an impact on the functioning of the smooth muscle, particularly uterine contractility, and play the role in preterm delivery [19].

The aim of this study was to determine if there are any changes in adiponectin and omentin levels in GDM patients who delivered at term and preterm and to evaluate whether adipokines can be useful as a clinical biomarker to predict subsequent preterm delivery.

## 2. Patients and Methods

One hundred and five women with GDM and 55 healthy pregnant women were included in the retrospective study, which was conducted in the Department of Obstetrics and Perinatology at Medical University of Lublin. The blood samples have previously been used to study the gestational diabetes mellitus pathogenesis in normal pregnant women and those with pregnancy complications. All patients signed informed consent to participate in the study, which had previously been approved by the Bioethical Review Board of the Medical University in Lublin. The study was performed according to the principles expressed in the Declaration of Helsinki.

The patients were divided into the following groups: (a) women with GDM who delivered at term (GD group: n=63); (b) women with GDM who had the symptoms of threatened preterm labor and delivered at term (TGD group: n=23); (c) women with GDM and spontaneous preterm birth (before 37 completed weeks of gestation) (PBGD group: n=19); (d) women with physiological pregnancy (PP group: n=55).

Exclusion criteria were as follows: multiple pregnancy, underlying disorders: prepregnancy diabetes mellitus, any form of hypertension, chronic renal disease, liver diseases, inflammation and infectious diseases, systemic lupus erythematosus, antiphospholipid syndrome, and intrauterine growth retardation.

The oral glucose tolerance test (OGTT) with 75 g of glucose according to WHO standards was performed in all women participating in the study and in the control group between 24th and 28th week of gestation. GDM was diagnosed on the basis of the following WHO criteria: fasting ≥92 mg/dL (5.1 mmol/L), at 1st hour ≥180 mg/dL (10.0 mmol/L), and at 2nd hour ≥153 mg/dL (8.5 mmol/L) [20].

Information about current and previous pregnancies, maternal and family anamnesis, maternal age, smoking status, and delivery outcomes was obtained using medical records.

Maternal prepregnancy body mass index (BMI) was calculated from maternal recall of weight prior to pregnancy and the height measured during the first visit before 8th week of gestation. BMI was recalculated at the time of blood sampling and at the time of delivery.

The blood samples for research tests were taken together with the samples for routinely performed laboratory tests. Serum levels of adiponectin and omentin were obtained at 24–28 weeks of gestation. The samples were allowed to clot for at least 30 minutes before centrifugation at 1000 G, which was continued for 20 minutes. Serum has been removed and then frozen at −70°C. The adiponectin concentration was measured by enzyme-linked immunosorbent assay technique (Human Adiponectin Elisa, High Sensitivity, BioVendor R&D Products, Czech Republic) as well as the omentin-1 concentration (Human Omentin-1 Elisa, BioVendor R&D Products, Czech Republic), according to the manufacturer's instructions.

## 3. Statistical Analysis

The obtained data were assessed by the one-way analysis of variance (ANOVA) followed by Tukey's* post hoc *test (Statistica, v. 12, StatSoft, Inc., Tulsa, OK, USA). Data distribution was assessed using Shapiro-Wilk test. Results with normal distribution are presented as the means ± standard deviation (SD); data with skewed distribution are presented as the medians with minimum and maximum values (Min./Max). To assess the correlation of the data, Pearson's and Spearman's correlation tests were performed. Herein, p < 0.05 was considered as a statistically significant difference. Univariable logistic regression models were used for calculations odds ratios (ORs) with 95% confidence intervals (CIs) predicting spontaneous preterm birth based on adiponectin and omentin concentrations.

## 4. Results

The studied groups did not differ with regard to the baseline descriptors: age, gravidity, estimated fetal weight, gestational age, and BMI at sampling. Expected differences in neonatal birth weight and gestational age at delivery were observed between PBGD and the other groups (Table 1). Prepregnancy BMI was higher in preterm birth group than the controls (24.8±2.5 versus 22.5±1.8 kg/m^2^, p<0.05) (Table 1).

In comparison with control group the median adiponectin concentrations were significantly lower in all GDM groups (10737 versus 8879; 7057; 6253 ng/ml, respectively; p<0.01). No significant differences in adiponectin levels between the GDM, preterm labor, and preterm birth groups were observed. However, there was a trend towards lower adiponectin level in preterm birth group (Table 2).

The median omentin concentrations were also significantly lower in all GDM groups in comparison with control group (469 versus 432; 357; 308 ng/ml, respectively; p<0.01) and no statistically significant differences between the GDM, preterm labor, and preterm birth groups were observed. As in the case of adiponectin, there was also a trend towards lower omentin level in preterm birth group (Table 2).

The strong correlations between adiponectin and omentin levels were observed in GD, TGD, PBGD, and PP groups (R=0.801, p<0.001; R=0.824, p<0.001; R=0.705, p<0.001; R=0.764, respectively; p<0.001) (Table 3).

Pairwise correlations between adiponectin and omentin-1 levels and clinical and demographic characteristics (maternal age; gravidity; prepregnancy, at sampling, and at delivery BMI; gestational age and estimated fetal weight at sampling; gestational age at delivery; baby's birth weight) were determined for all groups.

Adiponectin concentration was inversely correlated with prepregnancy, at sampling, and at delivery BMI in GD group (R=-0.828, -0.799, -0.822, respectively), TGD group (R=-0.868, -0.792, -0.758, respectively), PBGD group (R=-0.814, -0.703, -0.738, respectively), and PP group (R=-0.630, -0.539, -0.822, respectively) (Table 3).

As in the case of adiponectin, omentin levels were also inversely correlated with prepregnancy, at sampling, and at delivery BMI in GD group (R=-0.743, -0.712, -0.750, respectively), TGD group (R=-0.809, -0.553, -0.559, respectively), PBGD group (R=-0.800, -0.760, -0.756, respectively), and PP group (R=-0.04, -0.509, -0.430, respectively) (Table 4).

There was a correlation in GD group between prepregnancy BMI and gestational week at delivery (R=0.24, p<0.05), baby's birthweight (R=0.262, p<0.050), and maternal age (R=0.472, p< 0.001), but no correlations were found between BMI at sampling and at delivery and these parameters.

In the PBGD group the correlation between prepregnancy, at sampling, and at delivery BMI and maternal age was found (R=0.667, p<0.01; R=0.663, p<0.01; R=0.724, respectively; p<0.001).

In the univariable logistic regression model, no significant correlation between adiponectin and preterm birth was found.

We have found the correlation between the omentin concentration and the risk of preterm delivery. When we compared PBGD group versus PP group, omentin levels were inversely correlated with the risk of preterm birth (ß =-0.0188, p=0.0006, OR = 0.9814, 95% CI: 0.9710 - 0.9919). An increase of omentin level by 100 ng/ml decreases the possibility of preterm birth by almost 75%. When we compared PBGD group versus GDM group, omentin levels were also inversely correlated with the risk of preterm birth (ß =-0.0084, p= 0.017, OR= 0.9916, 95% CI: 0.9848 - 0.9985) and an increase of omentin level by 100 ng/ml decreases the possibility of preterm birth by almost 57%.

## 5. Discussion

In our study we have found the lower adiponectin levels in all GDM groups in comparison with uncomplicated pregnancy group, but we have not found significant differences in adiponectin levels between the women with GDM and GDM preterm labor and GDM preterm birth group. However, we noticed a trend towards lower adiponectin level in preterm birth group. In the univariable logistic regression model, no significant correlation between adiponectin and preterm birth was found.

Catalano et al. have noticed that the maternal adiponectin secretion progressively declines during the pregnancy. On the other hand, it has been published that the adiponectin levels during 1st, 2nd, and 3rd trimester were similar and were significantly higher than in postpartum period. Authors were of the opinion that despite the increased insulin resistance on the course of pregnancy, there were no significant changes in the adiponectin concentrations. This may signify that the regulation of the adiponectin release during gestation is altered [21]. It has not been confirmed whether there is adiponectin secretion from the placenta. Adiponectin can be found in fetal circulation after 24th week of gestation and the level tends to increase as pregnancy progresses. The fetal adiponectin levels have been observed to be significantly higher than those in the mother [22].

Adiponectin is adipokine, which in relation to GDM was the most widely studied. The data analyzing the adipokines levels in physiological and complicated pregnancies are ambiguous. It has been published that level of adiponectin was significantly lower in first and early second trimester in pregnant patient who later had diagnosed GDM [23]. Cortelazzi et al. have noticed that the levels of circulating adiponectin were lower in women with GDM as compared to patient in uncomplicated pregnancy [24]. A correlation between significantly decreased concentrations of adiponectin and beta cell impaired function in pregnant patient has been found, suggesting it may be an early marker of GDM development [25]. Mohammadi et al. analyzed the relationship of adiponectin levels to GDM and glucose intolerance. They measured the concentrations of serum adiponectin in GDM and healthy pregnant patients, who were screened between 24 and 28 weeks of pregnancy and compared the concentrations between the groups. Serum adiponectin concentrations were significantly lower in patients with GDM (5.10±2.15 ng/mL versus 7.86±3.52 ng/mL, p = 0.001) than in healthy pregnant women. These outcomes showed that serum concentrations of adiponectin were significantly lower in gestational diabetic women [26]. Doruk et al. investigated adiponectin levels in women with GDM and normal glucose tolerance (NGT) at 24-28 gestational weeks. Fasting serum adiponectin, glucose, and glycated hemoglobin (HbA1c) were determined in 88 pregnant women, 44 with GDM and 44 with NGT. Serum adiponectin levels were significantly reduced in GDM compared with the NGT group. The GDM group delivered significantly earlier than the NGT group. Adiponectin serum concentration was significantly reduced in patients with adverse outcomes and cesarean sections [27].

Although the signaling pathway of adiponectin (AMPK) has been described as a mechanism important to the myometrium, no data about the adiponectin effect on uterine contractility are available. A very limited number of articles focused on the adiponectin and preterm birth have been published. Mazaki-Tovi et al. examined pregnant women, who were divided into four groups: (1) uncomplicated pregnancy; (2) with an episode of preterm labor and intact membranes without intra-amniotic infection/inflammation (IAI) who delivered at term; (3) preterm labor without IAI who delivered preterm; and (4) preterm labor with IAI who delivered preterm. They analyzed levels of serum adiponectin multimers total, high-molecular-weight (HMW), medium-molecular-weight (MMW), and low-molecular-weight (LMW).) Lower median maternal serum concentration of total and HMW adiponectin was associated with preterm labor leading to preterm delivery or with an episode of preterm labor which does not lead to preterm delivery. Patients with preterm labor and IAI had the lowest median concentration of total and HMW adiponectin as well as the lowest median HMW/total adiponectin ratio. These findings confirm our results [28].

Vieira et al. in their multicentre study analyzed the factors, which could be predictors of uncomplicated pregnancy at birth. They examined obese (BMI≥ 30 kg/m^2^) pregnant women at 15^+0^ to 18^+6^ weeks of gestation to find sociodemographic, clinical, and biochemical factors. They identified them using multivariable logistic regression. These factors are multiparity, lower maternal age, systolic blood pressure, HbA1c levels, and elevated adiponectin levels (OR= 1.4(1.18-1.66); CI=95%) [29].

Kominarek et al. compared adipokines between experiencing preterm labor (PLT) and prior preterm deliveries (PTD). They analyzed serum levels of leptin, adiponectin, and resistin during pregnancy (three times: 23-34 weeks, 35-36 weeks, and at delivery). There were no significant differences in adipokines serum levels in patients with PLT and PTD, but they found correlation between body mass index (BMI) and levels of leptin and adiponectin. Researchers also compared levels of adipokines between patients who delivered at term and preterm. No significant differences were found, but levels of adiponectin were higher in preterm delivery group between 23 and 34 weeks and at delivery and lower between 35 and 36 weeks than in term delivery group [30].

Chervenak et al. tested mid-trimester amniotic fluid from 571 pregnant women for adiponectin, interleukin- (IL-) 6, IL-8, and *α*-amylase to identify adiponectin's associations with maternal parameters, pregnancy outcomes, and mediators in amniotic fluid. Adiponectin median levels were elevated in women with preterm premature rupture of membranes (pPROM) and women with an iatrogenic preterm birth (IPTB) in comparison to mothers with a term delivery (p= 0.0003). Higher median levels of adiponectin were also measured in patients whose infants developed fetal growth restriction and in patients whose babies had respiratory distress syndrome (p<0.0001). They also observed that adiponectin concentration was positively correlated with amylase level and inversely correlated with maternal body mass index. One of the limitations of study was ethnicity—most of the patients were Asian (309) [31].

In our study we have found the lower omentin levels in all GDM groups in comparison with uncomplicated pregnancy group, but we have not found significant differences in omentin levels between the women with GDM and GDM preterm labor and GDM preterm birth groups. However, we noticed a trend towards lower adiponectin level in preterm birth group. We have found the statistically significant inverse correlation between the omentin concentration and the risk of preterm delivery.

Omentin is a adipocytokine with insulin sensitizing effects in adipose tissues, but its function in vasculature is not clearly understood. Omentin has been described to participate in diverse pathophysiological processes. Yamawaki et al. have described that omentin inhibited TNF-*α*-induced cyclooxygenase-2 (COX-2) expression via activation of adenosine 5′-monophosphate-activated protein kinase (AMPK), which further activated the endothelial nitric oxide synthase (eNOS)/NO pathway [32]. Moreover, omentin induces a nitric oxide (NO)-mediated endothelium-dependent vasorelaxation, suggesting that omentin can take a part in the regulation of blood pressure through directly modulating contractile reactivity of blood vessels. However, the specific receptor for omentin has not been isolated yet [33]. The mechanisms by which the omentin can play a role in preterm birth are currently not very well understood. Nitric oxide relaxes also myometrial smooth muscle cells. Thus, we can speculate that similar relaxation effect of omentin could be found in the uterus. In preterm deliveries abnormal activation of proinflammatory cytokines was also described [34]. In* in vitro *studies Qi et al. have revealed that omentin-1 through the inhibition of COX-2 can also act as an anti-inflammatory agent [35]. Moreno-Navarrete et al. have noticed that omentin serum concentrations were inversely correlated with IL-6, TNF-*α*, and CRP levels and that can confirm the anti-inflammatory effects of this adipokine [36].

Omentin-1 is the major circulating form of omentin and very limited data on its concentrations in gestational diabetes mellitus are available. Similar omentin concentrations between GDM patients and pregnant controls were described and, on the other hand, Barker et al. demonstrated significantly decreased maternal circulating omentin concentrations in GDM compared with healthy pregnant patients. They have also found association of maternal obesity in pregnancy with decreased circulating omentin levels. Prospective studies of omentin as a predictor of gestational diabetes have not been published [37].

Several publications describing omentin in association with pregnancy have been published [37–39]. However, we have found only one article analyzing omentin-1 in relation to preterm birth. Šplíchal et al. compared levels of maternal omentin-1 and genetic variability between spontaneous preterm and term births. They evaluated omentin-1 levels and role of the omentin-1 Val109Asp (rs2274907) polymorphism in 30 pregnant patients with spontaneous preterm birth (sPTB) (16 with preterm premature rupture of membranes (pPROM) and 14 without pPROM) and 32 women with spontaneous term birth (sTB). Levels of maternal omentin-1 were significantly decreased in patients with sPTB, but there were no differences between women with and without pPROM. Scientists also observed no significant effect of the omentin-1 Val109Asp polymorphism on serum levels of omentin-1 [18]. They also revealed, in the univariate model, that the omentin-1 concentrations were negatively associated with sPTB occurrence (ß = -0.0029, OR = 0.9972, 95% CI: 0.9946 – 0.9998, p = 0.032) and calculate that if omentin-1 concentrations increase by 100 ng/ml the chance of sPTB occurrence decreases by approximately 25%. We have also found the same correlation between the omentin concentration and the risk of preterm delivery. Their findings confirm our omentin-1 results and what is also important, the examined population was also Caucasian.

Omentin-1 is mainly expressed in visceral adipose tissue. It has been described that omentin levels were negatively associated with the amount of visceral adipose tissue and BMI. We have found that omentin levels were inversely correlated with prepregnancy, at sampling, and at delivery BMI in all groups. In opposite to our results Šplíchal et al. did not find significant correlations between maternal omentin-1 levels and preconception BMI or BMI at delivery either in term births or in preterm births [18].

Barker et al. revealed that maternal omentin-1 levels were inversely correlated with fetal birth weight and fetal ponderal index [37]. In our study we did not observe any significant correlations between omentin concentrations and baby's birthweight.

The strong correlations between adiponectin and omentin serum levels were observed in all analyzed groups. To the best of our knowledge, this is the first study describing this association in pregnancy.

In presented study, in all analyzed groups, the negative correlation between adiponectin and omentin levels, and the prepregnancy, at sampling, and at delivery BMI were observed. Our observations partially confirm the results of the study presented by MacLachlan et al. who have found that the adiponectin levels are strongly related to BMI in pregnant women at 24-29th weeks of gestation [40]. Other report has revealed no correlation between the adiponectin concentrations and BMI in pregnant women [41]. Brandt et al. have found significant negative association between third trimester circulating maternal omentin levels and several maternal metabolic indices in a normal glucose tolerance population, including maternal BMI, insulin levels, and insulin resistance [42].

## 6. Conclusions

In conclusion, the adiponectin and omentin concentrations are decreased in all patients with gestational diabetes mellitus. The strong correlations between adiponectin and omentin levels were observed in all groups.

Our study suggests that circulating maternal adipokines may play a role in the pathophysiology of preterm birth. No significant differences in adiponectin and omentin levels between the GDM, preterm labor, and preterm birth groups were observed. However, there was a trend towards lower adiponectin and omentin levels in preterm birth group.

We have found the correlation between the omentin concentration and the risk of preterm delivery. Our findings suggest that omentin, rather than adiponectin, could be useful as a predictor of preterm birth in patients with gestational diabetes mellitus. On the other hand we know that further studies are required to confirm our outcomes. We hope that these results can help to identify pregnant patients, who need special care during pregnancy.

The limitations of the study also have to be acknowledged. All patients were Caucasian so the findings might not be applicable to other populations. The number of patients were relatively small. Thus, the analysis of outcomes may be underpowered. A larger scale research is recommended to address the limitations. However, we still continue our study, but in our opinion the findings were very interesting to show them on this stage of the study.

## Figures and Tables

**Table 1:** Characteristics of study population (mean and standard deviation).

	GDM (GD group) n=63	GDM and threatened preterm labor (TGD group) n=23	GDM and preterm birth (PBGD group) n=19	Uncomplicated pregnancy (PP group) n=55	p
Maternal age (years)	28.6 (5.1)	27.5 (4.0)	29.1 (4.0)	27.0 (4.5)	NS

Gravidity	2.12 (0.9)	2.38 (1.1)	2.0 (0.85)	2.0 (0.96)	NS

Gestational age at delivery (weeks)	39.3 (0.83)	39.4 (0.9)	32.3 (2.5)	39.4 (0.75)	p<0.001 PBGD vs GD; TGD; PP

Baby's birth weight (g)	3545 (406)	3465 (421)	1876 (161)	3410 (374)	p<0.001 PBGD vs GD; TGD; PP

Pre-pregnancy BMI (kg/m^2^)	24.5 (2.6)	23.8 (2.4)	24.8 (2.5)	22,5 (1.8)	p<0.05 PBGD vs PP

BMI at sampling (kg/m^2^)	26.8 (2.2)	26.4 (1.6)	26.7 (1.98)	25.0 (1.5)	NS

BMI at delivery (kg/m^2^)	28.8 (2.3)	28.7 (1.4)	28.1 (2.0)	27.2 (1.3)	p<0,05 GD vs PP

Estimated fetal weight at sampling (g)	980 (206)	898 (174)	875 (161)	981 (152)	NS

Gestational age at sampling (weeks)	27.0 (1.5)	26.3 (1.3)	26.1 (1.2)	26.9 (1.3)	NS

**Table 2:** Median (range) concentrations of adiponectin and omentin in blood sampling.

	GDM (GD group) n=63	GDM and threatened preterm labor (TGD group) n=23	GDM and preterm birth (PBGD group) n=19	Uncomplicated pregnancy (PP group) n=55	p
Adiponectin ng/ml	8879 (3832-16348)	7057 (3429- 12907)	6253 (3607- 10801)	10737 (5294- 18842)	p<0.01 PP vs GD; TGD; PBGD

Omentin ng/ml	432 (195-687)	357 (258-588)	308 (244-519)	469 (307-799)	p<0.01 PP vs GD; TGD; PBGD

**Table 3:** Correlation between adiponectin and omentin, maternal age, gravidity, gestational age at delivery, baby's birthweight, and BMI.

	GDM		GDM and threatened preterm labor		GDM and preterm birth		Uncomplicated pregnancy	
	Adiponectin		Adiponectin		Adiponectin		Adiponectin	

	R	p	R	p	R	P	R	p

Omentin	0.801	p<0.001	0.824	p<0.001	0.705	p<0.001	0.764	p<0.001

Maternal age (years)	- 0.365	p<0.01	-0.257	NS	-0.483	p<0.05	-0.187	NS

Gravidity	-0.352	p< 0.01	-0.039	NS	-0.003	NS	-0.093	NS

Gestational age at delivery (weeks)	-0.173	NS	0.022	NS	-0.211	NS	-0.024	NS

Baby birthweight (g)	-0.138	NS	-0.058	NS	-0.458	p<0.05	0.083	NS

Pre-pregnancy BMI (kg/m^2^)	-0.828	p<0.001	-0.868	p<0.001	-0.814	p<0.001	-0.630	p<0.01

BMI at sampling (kg/m^2^)	-0.799	p<0.001	-0.792	p<0.001	-0.703	p<0.01	-0.539	p<0.01

BMI at delivery (kg/m^2^)	-0.822	p<0.001	-0.758	p<0.001	-0.738	p<0.001	-0.447	p<0.01

Gestational age at sampling	-0.053	NS	0.082	NS	-0.476	p<0.05	0.011	NS

Estimated fetal weight at sampling	-0.003	NS	-0.049	NS	-0.458	p<0.05	0.018	NS

GDM: gestational diabetes mellitus.

BMI: body mass index.

R: Spearman's correlation coefficient.

p: statistical significance.

NS: statistically not significant.

**Table 4:** Correlation between omentin and adiponectin, maternal age, gravidity, gestational age at delivery, baby's birthweight, and BMI.

	GDM		GDM and threatened preterm labor		GDM and preterm birth		Uncomplicated pregnancy	
	Omentin		Omentin		Omentin		Omentin	

	R	p	R	p	R	P	R	p

Adiponectin	0.801	p<0.001	0.824	p<0.001	0.705	p<0.001	0.764	p<0.001

Maternal age (years)	-0.358	p<0.05	-0.029	NS	-0.336	NS	-0.187	NS

Gravidity	-0.290	p<0.05	-0,091	NS	-0.016	NS	-0.091	NS

Gestational age at delivery (weeks)	-0.267	p<0.05	-0.111	NS	-0.150	NS	-0.075	NS

Baby birthweight (g)	0.001	NS	-0.061	NS	-0.152	NS	-0.048	NS

Pre-pregnancy BMI (kg/m^2^)	-0.743	p<0.001	-0.809	p<0.001	-0.800	p<0.001	-0.604	p<0.01

BMI at sampling (kg/m^2^)	-0.712	p<0.001	-0.553	p<0.01	-0.760	p<0.001	-0.509	p<0.01

BMI at delivery (kg/m^2^)	-0.750	p<0.001	-0.557	p<0.01	-0.756	p<0.001	-0.430	p<0.01

Gestational age at sampling	-0.067	NS	-0.013	NS	-0.310	NS	-0.048	NS

Estimated fetal weight at sampling	0.001	NS	-0.061	NS	-0.200	NS	-0.030	NS

GDM: gestational diabetes mellitus.

BMI: body mass index.

R: Spearman's correlation coefficient.

p: statistical significance.

NS: statistically not significant.

## Data Availability

The data used to support the findings of this study are included within the article.
